# Light Converts Endosymbiotic Fungus to Pathogen, Influencing Seedling Survival and Niche-Space Filling of a Common Tropical Tree, *Iriartea deltoidea*

**DOI:** 10.1371/journal.pone.0016386

**Published:** 2011-01-31

**Authors:** Patricia Álvarez-Loayza, James F. White, Mónica S. Torres, Henrik Balslev, Thea Kristiansen, Jens-Christian Svenning, Nathalie Gil

**Affiliations:** 1 Department of Plant Biology and Pathology, School of Environmental and Biological Sciences, Rutgers University, New Brunswick, New Jersey, United States of America; 2 Center for Tropical Conservation, Nicholas School of the Environment, Duke University, Durham, North Carolina, United States of America; 3 Ecoinformatics and Biodiversity Group, Department of Biological Sciences, Aarhus University, Aarhus, Denmark; 4 Universidad Nacional San Antonio de Abad, Cusco, Peru; University of Wisconsin – Madison, United States of America

## Abstract

Pathogens are hypothesized to play an important role in the maintenance of tropical forest plant species richness. Notably, species richness may be promoted by incomplete filling of niche space due interactions of host populations with their pathogens. A potentially important group of pathogens are endophytic fungi, which asymptomatically colonize plants and are diverse and abundant in tropical ecosystems. Endophytes may alter competitive abilities of host individuals and improve host fitness under stress, but may also become pathogenic. Little is known of the impacts of endophytes on niche-space filling of their hosts.

Here we evaluate how a widespread fungal endophyte infecting a common tropical palm influences its recruitment and survival in natural ecosystems, and whether this impact is modulated by the abiotic environment, potentially constraining host niche-space filling. *Iriartea deltoidea* dominates many wet lowland Neotropical forests. *Diplodia mutila* is a common asymptomatic endophyte in mature plants; however, it causes disease in some seedlings. We investigated the effects of light availability on *D. mutila* disease expression.

We found *I. deltoidea* seedlings to preferentially occur under shady conditions. Correspondingly, we also found that high light triggers endophyte pathogenicity, while low light favors endosymbiotic development, constraining recruitment of endophyte-infested seedlings to shaded understory by reducing seedling survival in direct light. Pathogenicity of *D. mutila* under high light is proposed to result from light-induced production of H_2_O_2_ by the fungus, triggering hypersensitivity, cell death, and tissue necrosis in the palm. This is the first study to demonstrate that endophytes respond to abiotic factors to influence plant distributions in natural ecosystems; and the first to identify light as a factor influencing where an endophyte is placed on the endosymbiont–pathogen continuum. Our findings show that pathogens can indeed constrain niche-space filling of otherwise successful tropical plant species, providing unoccupied niche space for other species.

## Introduction

Pathogens and other natural enemies have long been hypothesized to play an important role in the local maintenance of species diversity, especially in species-rich tropical forests [1], [2], [3]. Notably, it has recently been proposed that species richness may be promoted by incomplete filling of niche space due localized dynamic coevolutionary interactions of populations with their pathogens or other natural enemies [4]. However, direct empirical evidence for such host-pathogen interactions and their ecological importance remain scarce (e.g., [5], [6], [7]). A potentially important group of pathogens are endophytic fungi, which asymptomatically colonize plants [8] and are diverse and abundant in tropical ecosystems [9]. These organisms may be pathogenic and/or mutualistic, depending on the circumstances [10]. The variable virulence of the endophyte, the host defense response, and environmental conditions constitute the disease triangle [11], ‘or disease/mutualism triangle’, and could be influencing the where an endophyte is placed on the endosymbiont-pathogen continuum. This has never been investigated though. Suboptimal environmental conditions may stress the host defense status, resulting in disease [12]. Several studies have shown that under conditions of stress, inoculation of endophytes into plant tissues [13] often resulted in disease symptoms (necrosis or chlorosis) and/or growth inhibition of the host. Additionally, other studies have focused on evaluating how endophytes alter competitive abilities of host individuals and improve host fitness under abiotic or biotic stress [14], [15]. However, none have looked for or identified environmental factors that alter the behavior of endophytes in natural ecosystems, their relationships to hosts, and the ecological implications for the niche-space filling (spatial and environmental patterns of distribution and abundance) of host plant species.

The palm *Iriartea deltoidea* is one of the most dominant tree species in wet lowland and premontane tropical forests of western Amazonia [16], [17], [18] and the Chocó- and Central American region [19], [20]. In contrast to most large palms [21], this species does not depend on large forest gaps for recruitment [22], perhaps related to its peculiar growth strategy. This palm undergoes ontogenetic transitions in leaf morphology. Young seedlings produce 3 to 6 round, simple leaves. Subsequent leaves are longer and compound, carrying increasing numbers of pinnae [23]. However, the inordinate success of *I. deltoidea* in wet New World tropical forests remains an enigma and cannot be explained by morphological attributes such as fruit size or height [19], [24].

*Diplodia mutila* is an endophytic/pathogenic fungus infecting *I. deltoidea* [25], with approximately 88.4% of the adult palms being infected, 45.1% of newborn seedlings, and 63.2% of 1-year old seedlings [29]. *Diplodia mutila* and related species have been reported as endophytes or latent pathogens for several plant species worldwide [27], [28], [29]. The fungus is an asymptomatic endophyte in *I deltoidea* mature plants, and disease and mortality are expressed in some seedlings, while others remain disease free. The infection pattern of *D. mutila* does not correspond with the one described in the J-C hypothesis [1], [2]. Infected plants are found near and far away from parental trees [26]. Here we investigate if the widespread endophytic fungal pathogen *D. mutila* influences survival and recruitment patterns of its host plant the common tropical palm, *I. deltoidea* in natural ecosystems and whether this impact is modulated by the abiotic environment, potentially affecting host niche-space filling. Additionally we investigated whether *D. mutila* alters the competitive ability of *I. deltoidea* under biotic stress (insect attack).

## Results

### Light conditions for *I. deltoidea* seedlings

Seedlings of *I. deltoidea* preferentially occur under shady conditions. Extensive sampling at two sites in western Amazonia showed that more than 91% of *I. deltoidea* seedlings were found in understory conditions. The total number of seedlings in the Northeastern Peru transects was 660, 94% of these seedlings were located at understory conditions (canopy scope <5). The negative correlation between the number of *I. deltoidea* seedlings and canopy openness in the 102 transects (Spearman *r* = −0.117, *P*<0.05) was estimated using *n* = 280 5×5 m subplots with at least one *I. deltoidea* seedling. Statistical significance was assessed as a one-tailed test and correcting for spatial autocorrelation using Dutilleul's approach for computing the geographically effective degrees of freedom  = 227 [30]. The total number of seedlings in the Southeastern Peru plots was 518 seedlings (less than 25 cm), 91% seedlings were located in dense understory (∼55–120±15 µmol m^−2^ s^−1^).

### High light availability increases plant disease development

**Figure 1:**
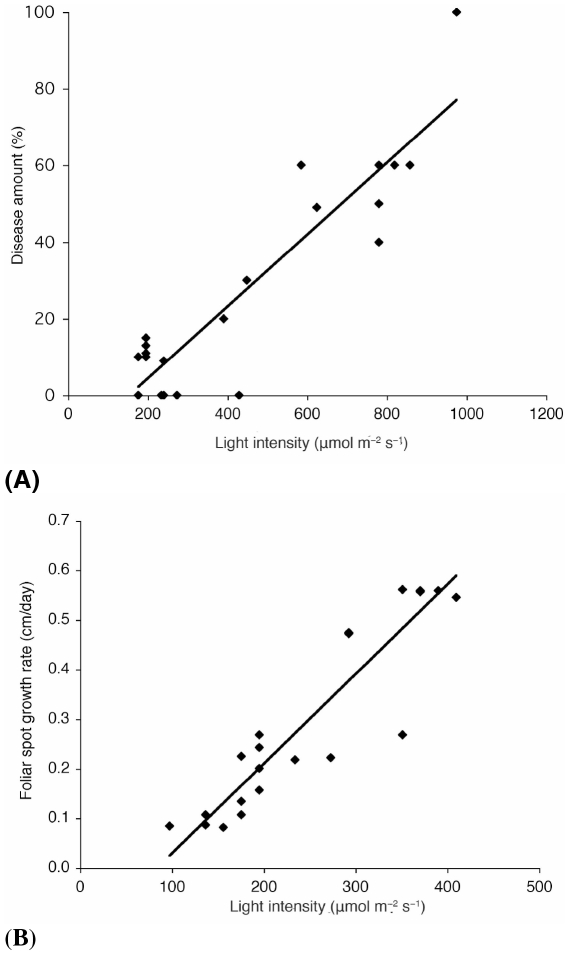
Higher light intensities increased disease development produced by *Diplodia mutila*. (**A**) For young seedlings with 2 leaves or less there was a significant interaction between amount of infection (% of *D. mutila* foliar spots in *Iriartea deltoidea* leaves) and light level (*F*_1,22_ = 55.4, *P* = 0.0001**, *r* ^2^ = 0.73). (**B**) The diametric growth rate of the foliar spots produced by *D. mutila* was higher at higher light conditions (ANOVA, *F*_1,22_ = 93.26, *P* = 0.0001**, *r* ^2^ = 0.816).

We found that *D. mutila* is beneficial to *I. deltoidea* under understory conditions, but strongly reduces *I*. *deltoidea*'s capacity for recruitment in high-light forest gaps. Additionally, the foliar necrotic spot symptoms produced by *D. mutila* appeared more frequently in seedlings and juveniles that grew in gaps or diffuse open canopy conditions: Plants with visible symptoms caused by *D. mutila* received significantly higher illumination, 408.3±17.3 µmol m^−2^ s^−1^ than plants with no visible symptoms receiving lower illumination, 208.2±6.1 µmol m^−2^ s^−1^, (mean ± SE, *t* test, n = 808, *P*<0.0001). Disease development was faster and more lethal in seedlings with two leaves or less when exposed to higher light conditions (*F*_1,22_ = 55.4, *P* = 0.0001, *r^2^* = 0.73) (Fig. 1A). These results are consistent with the landscape recruitment patterns of *I. deltoidea* mentioned above. Ontogenic or age-related resistance may be responsible for differences in disease expression between seedlings in different stages of development [31]. An additional experiment showed that pathogenicity of *D. mutila* increased with light availability. We inoculated 22 healthy 6-month old *I. deltoidea* seedlings (no foliar spots or insect marks) with *D. mutila*, following inoculation procedures from previous studies [25]. Foliar spots produced by *D. mutila* had a higher growth rate and mortality was greater and faster at higher light availability (*F*_1,22_ = 93.26, *P* = 0.0001, *r* ^2^ = 0.816) (Fig. 1B).

**Figure 2:**
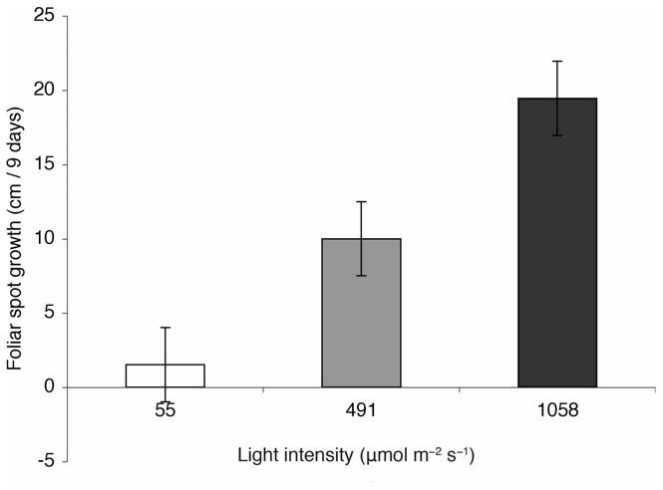
Increased light availability switched the endosymbiotic phase of *D mutila* to its pathogenic phase. Young seedlings that were colonized with endophytic *Diplodia mutila* showed faster growth rates of diameter of foliar spots (cm) caused by the pathogenic phase of *D. mutila* at higher light intensities (∼1058±23 µmol m^−2^ s^−1^) than seedlings under shaded conditions (∼55±15 µmol m^−2^ s^−1^) (n = 30, *t* test, *P* = 0.0001**). There were also significant differences of foliar spot growth rates among plants growing in the greenhouse (∼491±34 µmol m^−2^ s^−1^) and plants growing under shaded conditions (n = 30, *t* test, *P* = 0.024*). Foliar spot growth rates among plants growing in the greenhouse were lower than plants growing under high light intensities (n = 30, *t* test, *P* = 0.013*), (Tukey Kramer HSD test, ANOVA, *F*_3,30_ = 12.62, *P* = 0.0001**).

Using transplant experiments we demonstrated that increased light availability switched the endosymbiotic phase of the fungus to its pathogenic phase. In a greenhouse experiment diametric growth rate of foliar spots produced by *D. mutila* was higher in full sun conditions (mean 19.5±2.5 SE cm/day) than in reduced light (10.0±2.5 cm/day) and full shaded conditions (0.52±2.5 cm/day; analysis of variance (ANOVA), *F*_3,30_ = 12.62, *P* = 0.0001; Fig. 2). *Diplodia mutila*-induced seedling mortality in plants exposed to full sun was 80% after ten days. Seedlings under shaded conditions had 10% mortality and seedlings in the greenhouse had 40% mortality.

**Figure 3:**
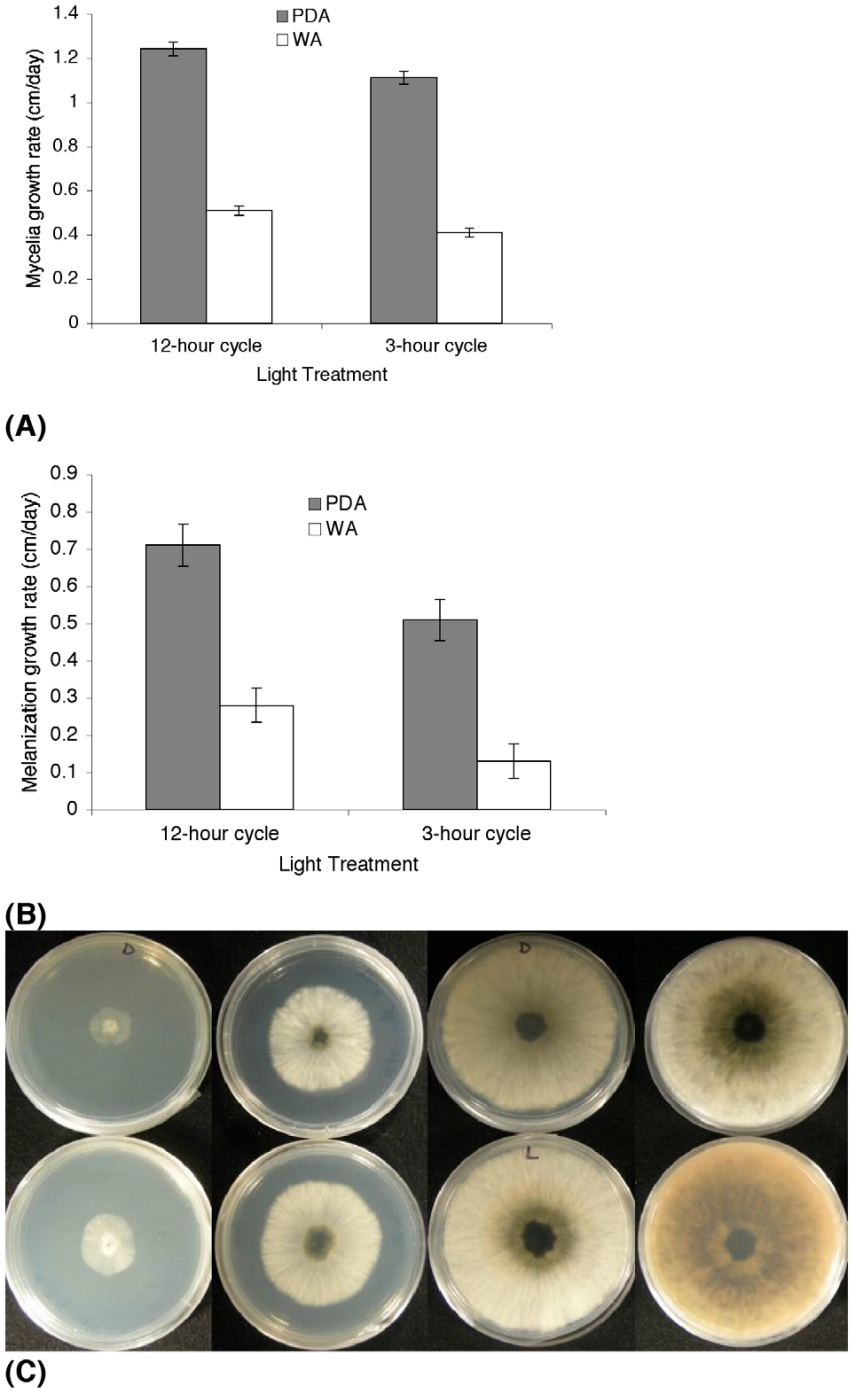
Fungal growth variation of *Diplodia mutila* under different light treatments and two different media. (**A**) Mycelial radial growth of *Diplodia mutila* on Potato Dextrose Agar (PDA) was faster under a 12-hour cycle than the 3-hour cycle: 1.25 (±0.03) cm/day vs. 1.11 (±0.03) cm./day (n = 12, *t* test, *P*>0.018*). On Water Agar (WA) the average radial growth rate per day of the colony mycelium was 0.51 cm/day under a 12-hour light cycle; while under a 3-hour light cycle the average growth rate of the colony mycelium was significantly lower, at 0.41 cm/day after 7 days (n = 12, *t* test, *P*>0.004*). (**B**) Colonies grown in PDA under the 12-hour light cycle had a more rapid melanization of the central area of the colony (∼0.71 cm/day) than colonies exposed to 3-hours of light (∼0.5 cm/day) (n = 12, *t* test, *P*>0.022*). Similar results were obtained for colonies growing in WA (n = 12, *t* test, *P*>0.026*). (**C**) Melanization of colonies of *D. mutila* growing in PDA observed on 4 days (from left to right). Rate of melanization was reduced in the 3-hour cycle treatment (above). Faster melanization was observed in cultures maintained in a 12-hour light cycle (below).

Laboratory assays showed that fungal growth (measured as diameter of mycelial colonies or as density of mycelium comprising colony) was greater when a 12-hr alternating light-dark cycle was provided than when periods of light were restricted to 3 hours. On Water Agar medium (WA) the average growth rate per day of the colony mycelium for five days was higher under a 12-hour light cycle (mean 0.52±0.03 SE cm/day) than under a 3-hour light cycle (0.38±0.03 cm/day, *t* test, n = 12, *P*<0.004). On Potato Dextrose Agar medium (PDA) the average growth rate per day of the colony mycelium was faster and also higher under the longer light period (mean 1.25±0.01 SE cm/day), compared to 1.11±0.11 cm/day for the 3-hour photoperiod (*t* test, n = 12, *P*<0.018) and the mycelium was notably denser with more aerial mycelium (Fig. 3A). We recorded greater melanization of mycelium in colonies exposed to the longer light period. This was especially evident in colonies grown on PDA: Colonies grown in PDA under the 12-hour light cycle had significantly faster growth of the central melanized area (mean 0.71±0.05 SE cm/day) than colonies exposed to the 3-hour light treatment (0.5±0.05 cm/day, *t* test, n = 12, *P*<0.022; Fig. 3B, 3D). The absorption spectrum of methanolic extracts of *D.mutila* cultures showed a characteristic peak in the UV region of 200–260 nm [32]. HPLC chromatograms of methanolic extracts of light and dark grown culture extracts showed that light grown cultures have at least twice the amount of melanin than dark grown cultures. Melanization of mycelium has been linked to enhanced virulence in numerous plant and animal pathogenic fungi [33]. Similar significant results were obtained for colonies growing in WA medium (Fig. 3B).

**Figure 4:**
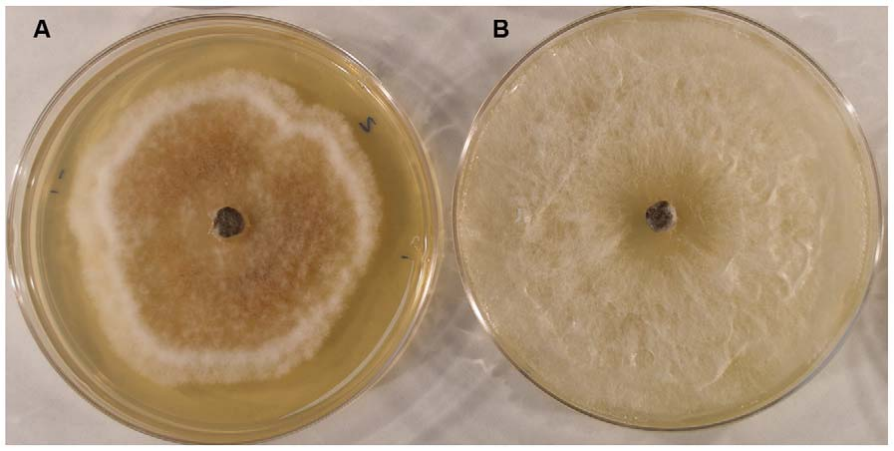
*In vitro* hydrogen peroxide production of *Diplodia mutila* on Potato Dextrose Agar (PDA). (**A**) Hydrogen peroxide (orange pigmentation) accumulation in culture grown under light at 30°C treatment. (**B**) Culture grown under dark at 30°C treatment, showing no or minimal accumulation of hydrogen peroxide.

Colonies grown on PDA under high light conditions showed higher hydrogen peroxide production than those grown under low light conditions as evidenced by staining using the DAB/peroxidase H_2_O_2_ assay (Fig. 4). Hydrogen peroxide has been shown to be a signal molecule for plants that triggers hypersensitivity cell and tissue death [34].

### Distribution of seedlings affected by biotic stress (stem borers)

We found that stem borers (order Coleoptera) are the main mortality cause of *I. deltoidea* seedlings. After 50 days, 9% of the marked plants had died. Stem borers were the cause of 73% of the total palm mortality after 50 days, while *D. mutila* (in its pathogenic phase) caused just 2% of the mortality. After 150 days, stem borers induced 43% of mortality, while *D. mutila* (pathogenic phase) was responsible for a mere 0.1% of the mortality. We did not find any relationship between light availability and endophyte presence.

The proportion of plants affected by stem borers within the first 2.5 m near the parental tree was significantly higher than proportions in the other 4 annuli far away from the parental tree, in the first census: After ∼7 days, stem-borer infection was 8%±0.01% (mean ± SE) in the inner annulus, and 6%±0.01%, 4%±0.01%, 3%±0.01%, and 3%±0.01% in the outer annuli (going outwards) (One-way ANOVA, *F_4,50_* = 2.65, *P*>0.045*). After ∼50 days, the same numbers were 10%±0.01%, 4%±0.01%, 1%±0.01%, 2%±0.01%, and 0.7%±0.01% (One-way ANOVA, *F_4,50_* = 4.28, *P*>0.0051*).

### Protection from insect pests

We found evidence that *D. mutila* benefits its host plants by enhancing resistance to herbivory by some insects. Field surveys in the ten surveyed plots, showed that insect herbivory (stem borers) decreased with increasing incidence of *D. mutila* infection. Plots with few *D. mutila*-infested *I. deltoidea* plants had higher incidence of stem-borer mortality, whereas plots with higher incidence of plants colonized by *D. mutila* had lower rates of stem-borer- induced mortality (ANOVA, *F*_1, 10_ = 18.49, *P* = 0.0026, *r^2^* = 0.69). Additional feeding experiments employing *I. deltoidea* fruits and PDA media (Potato Dextrose Agar) colonized by *D. mutila* showed that adults of the beetle *Coccotrypes* sp., and two unidentified species of larvae of the order Coleoptera avoided consumption of fruits and PDA colonized by *D. mutila*. Beetles consistently preferred PDA and avoided *D. mutila* infested PDA, mean 4.8 beetles ±0.14 SE versus 1.4 beetles±0.14 (Repeated measures ANOVA, *F_4, 456_* = 160.13, *P* = 0.0001**). Similar results were also obtained when the experiment was performed using *Coccotrypes* sp. adults and *I. deltoidea* fruits instead of PDA.

## Discussion

**Figure 5:**
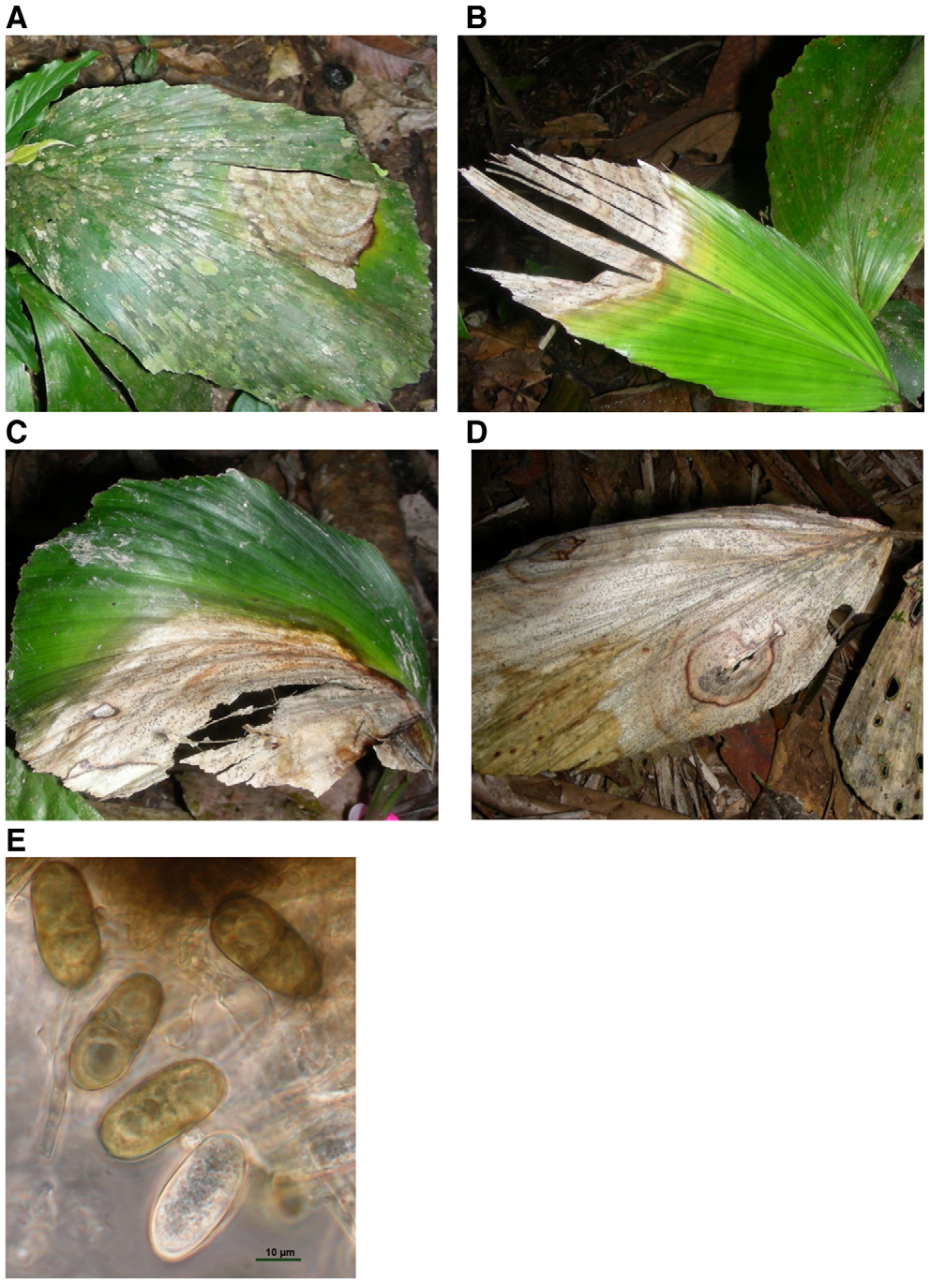
Foliar spots in *Iriartea deltoidea* caused by *Diplodia mutila*, at different infection stages. (**A**) Leaf spot infection for a plant with 2 leaves and one spot covering less than 20% of the leaf (**B**) A plant with two leaves and with a spot covering ∼40% of one leaf (**C**) A plant with two leaves and with the two foliar spots covering 50% of both leaves (**D**) Foliar spots covering the entire plant, representing 100% of infection. These plants died after 15 to 31 days. (**E**) *Diplodia mutila* pycnidia produced slowly maturing, non-striate, brown, 1-septate conidia measuring 26–28×15–20 µm. Liquid conidial darkening and septation was recorded to take place after discharge.

*Diplodia mutila* is frequently an asymptomatic endophyte in seeds, fruits, leaves of healthy juvenile and mature plants of *I. deltoidea* (88.4% infection of adult plants [29]) but can also act as a pathogen, causing mortality in young *I. deltoidea* seedlings after 5 to 16 days of infection and producing foliar spots in adult plants [25] (Fig. 5A–D). In the pathogenic phase, *D. mutila* forms pycnidia, flask-shaped asexual structures that exude masses of uni-cellular to bi-cellular, hyaline to brown conidia [35] (Fig. 5E). In its endophytic phase the fungus exists only as mycelium within tissues of the host's leaves, stems and seeds [8]. An endophyte life cycle that includes both vertical transmission through host seeds and infective spread using fungal spores (horizontal transmission) should provide enhanced dispersal potential over fungal life cycles that involve only a single transmission pathway.

We found that high light triggers pathogenicity of the fungus, while low light favors endosymbiotic development, constraining recruitment of endophyte-infested seedlings to the shaded understory by limiting survival of seedlings in direct light, i.e., constraining the niche-space filling [4] of the otherwise highly successful *I. deltoidea*. *In vitro* studies show that light induces the fungus to produce melanin and secrete hydrogen peroxide. Hydrogen peroxide is a plant signal molecule that induces hypersensitivity cell death response in plants [34]. In alternating light/dark cycle experiments, radial growth of *D. mutila* was also enhanced by longer periods of light, and faster growth may contribute to disease expression by the fungus.

Laboratory experiments indicate that mycelial melanin and hydrogen peroxide production increase with light exposure. Melanin correlates with increased production of reactive oxygen species (ros) in pathogens and is often associated with increased virulence to hosts. Melanin is generally believed to be an antioxidant defense in fungal pathogens [32], and appears be produced in cultures of *D. mutila* that show enhanced hydrogen peroxide production. We suggest that higher light intensity increases fungus virulence to plants by triggering the hypersensitivity cell death response in plants [34]. An explanation for the relationship between light and H_2_O_2_ production may be found in the glyoxylate cycle, where H_2_O_2_ is produced by the fungus. The activity of the glyoxylate cycle is known to be enhanced by exposure to light [36].

Regardless of the precise explanation, it is apparent that *D. mutila*-colonized *I. deltoidea* seedlings survive better under closed canopy conditions due to the effect of light in triggering the pathogenic phase of the endophyte. When these plants become older seedlings, the pathogen does not seem to affect plant performance even at high light availability and, additionally, may confer other advantages to these plants, i.e., defensive mutualism [37]. Endophytes in many plants have been shown to provide hosts with increased herbivore and/or environmental stress resistance [14], [15], [38]. In the case of the *D. mutila*-palm association we found that infection correlated with reductions in the insect herbivory (stem borer attack) on the palm and thus probably improves host fitness. This effect is all the more important since stem borers were the main mortality agent for *I. deltoidea* seedlings, causing much more mortality than pathogenic *D. mutila* infections. Importantly, mortality rates after 50 and 150 days were higher next to fruiting trees and mostly produced by stem borers. Most young seedlings infested with stem borers were located in the vicinity of the fruiting adults, under closed canopy conditions. Hereby, the stem borer attack pattern follows the Janzen-Connell (J–C) hypothesis, which predicts high mortality of seeds and juvenile plants near parent trees, a process that facilitates the establishment of heterospecifics and therefore promote maintenance of plant diversity [1], [2]. Hereby, *D. mutila* should reduce negative density—dependence in the recruitment of *I. deltoidea*. Adding complexity to this finding, the case of *D. mutila* furthermore demonstrates that the environment can drastically modulate how an endosymbiotic fungus affects fitness of its host.

While pathogens and other natural enemies may potentially play crucial roles in driving diversification dynamics and maintaining species diversity [1], [2], [3], [4], we still have a very limited understanding of the actual roles played by pathogens in natural ecosystems. Our findings show that host plant characteristics such as age, light-dependent pathogenicity and virulence of an endophyte-pathogen (i.e., *D. mutila)* are intrinsically connected, influencing patterns of host seedling survival, potentially driving small-scale host species distribution patterns as well as its abundance at larger scales. Notably, *D. mutila* limits niche-space filling in an otherwise highly successful plant species, providing niche space for other species [4]. The resistance to insect predators such as stem and seed borers conferred by *D. mutila* may allow *I. deltoidea* to escape the otherwise potentially high intraspecific density- and distance-dependent mortality [1], [2], [3] and recruit - in the shady understory - near adult trees. Hence, while *D. mutila* constrains the niche-space filling of *I. deltoidea*, it may at the same time also contribute to this species' extraordinary abundance in many Neotropical wet forests [16], [17], [18], [19], [20]. It seems clear that patterns of plant abundance and the mechanisms maintaining tropical forest biodiversity may be the result of a more complex interplay between the abiotic environment and biotic interactions than previously thought, with pathogens playing an important role. However, much more research is needed before we can fully evaluate the extent to which microorganisms influence plant populations and community assembly in tropical ecosystems.

## Materials and Methods

### Demographic censuses

In Northeastern Peru [cf. 39] we arbitrarily placed 102 transects (5×500 m, divided in 5×5 m subunits) located in mature primary tropical rain forest within 300 km of Iquitos, Peru (excluding transects located in secondary forests, white sand soils, steep topographical conditions and human disturbed forests). Sites in Southeastern Peru were located at Cocha Cashu, (CCBS) [40] and Los Amigos, (LABS) [41]. Ten plots were established in May 2007, in primary floodplain forest, with similar floristic composition and topographic characteristics. Five of plots were located at CCBS and five at LABS. Nine plots measured 900 m^2^ and one plot at CCBS measured 2.25 ha. In each plot all *I. deltoidea* plants were tagged with numbered plastic tags and mapped in an X - Y coordinate system. The total number of plants located in the ten plots was 1068: 63 fruiting adults, 518 seedlings and 487 were considered juveniles-adults (non-fruiting). We measured height of the tallest photosynthetic leaf (cm) and number of leaves and diameter of foliar spots caused by *D. mutila* (cm) for all seedlings. The amount of disease was calculated by dividing the diameter of the foliar spot by the diameter of the affected leaf and expressed as ‘% disease’. Disease development over a period of 150 days was calculated by subtracting the % disease in the initial estimate from the final estimate.

### Distribution of seedlings affected by biotic stress (stem borers)

Plants damaged and killed by epicotyl borers, such as caterpillars, beetle larvae or crickets, were considered as “damaged by stem borers”. Plants located in the Southeastern Peru plots were monitored for presence/absence of *D. mutila* and stem borers, three times after initial establishment (7, 50 and 150 days). In each plot, the minimum distances from all seedlings to the nearest *I. deltoidea* fruiting plant were computed using the coordinates of the labeled plant under consideration and the coordinates of the nearest fruiting tree within the plot. We surveyed seedlings in 5 concentric 2.5 m annuli centered on a focal fruiting tree. The number of seedlings affected by stem borers and *D. mutila* was tallied for each 2.5 m annulus and then divided by the total number of plants located in the selected annulus to yield proportions. One-way ANOVA was used to compare diseases and mortality proportions among plots for each distance annulus (Tukey's HSD used to contrast means).

### Light availability measurement

In Northeastern Peru light availability was measured using the canopy scope methodology [42]. In Southeastern Peru light availability was estimated above the tallest photosynthetic leaf of each *I. deltoidea* seedling, using the average value of light intensity over the leaf with a light meter (Environmental Concepts Plant Light Intensity Meters, LIM2500, USA). The average value was obtained from three measurements over each plant at 6 am, 12 pm and 5 pm for three consecutive days.

### Transplant experiments

We transplanted 30 *I. deltoidea* seedlings from one plot at Cocha Cashu where adults, juveniles, seeds and fruits were colonized by *D. mutila*. Ten seedlings were transplanted to shade conditions, ∼55±15 µmol m^−2^ s^−1^, ten to a reduced light environment in a greenhouse, ∼491±34 µmol m^−2^ s^−1^, and ten to full sun exposure, ∼1058±23 µmol m^−2^ s^−1^. All seedlings had two leaves and did not have any visible disease symptoms produced by *D. mutila* or any other foliar spot. Light availability was measured three times a day (6 am, 12 pm and 5 pm) for a period of ten days and all disease symptoms and insect damage were recorded and measured daily. The average daily temperature in the understory and full sun conditions was 23±3°C and 26±5°C in the greenhouse.

### In vitro deterrence experiment

On December 2007, ∼370 *Coccotrypes* sp. beetles and larvae were extracted from more than 100 fruits and seeds of *I. deltoidea*. In a Petri plate (60×15 mm, Fisher Scientific Co. Canada) we placed two 1-cm^2^ of PDA (Potato Dextrose Agar) and two 1-cm^2^ of PDA infested with *D. mutila*, covered with squares of non-acidic paper to simulate dark conditions found inside seeds and fruits. PDA was replaced everyday for the duration of the experiment to avoid contamination of non-infested PDA by *D. mutila*. We set up 12 repetitions following this procedure. Six to ten beetles were released in the each Petri plate and monitored daily for 8 to 12 days.

### Laboratory assays

To assess the effect of light on the fungus, laboratory observations were made on mycelial growth in Water Agar (WA) and Potato Dextrose Agar (PDA). Two photoperiod treatments were employed for five days with six *D. mutila* samples per treatment. The first treatment consisted of 12-hour cycle of darkness and 12-hour cycle of white light (fluorescent, 100±10 µmol m^−2^ s^−1^), while the second consisted of 21-hour cycle of darkness and 3-hour cycle of light for five days, (6 repetitions per treatment, constant temperature for all treatments was 24°C).

### Analysis of methanolic extracts of *D. mutila* for *in vitro* production of melanin

Methanolic extracts of culture discs from *D. mutila* grown at room temperature and 30°C in dark and light conditions were analyzed by HPLC [34]. Fungal cultures were extracted in methanol 80% at day 10 of the experiment. Samples were concentrated using a rotovapour (model 11, Buchi, Flawil, Switzerland) at 45°C and dried in speed vacuum (Savant PSD 2010 Speed Vac Concentrator, Thermo). Dried samples were dissolved in 1 ml of methanol 80% with add of vortex and sonication, and filtered using 0.45 µm Nylon (CostarR) filters held in a 2.0 ml polypropylene tube and centrifuged at 5000 rpm for 45 sec before analysis. Sample analysis was conducted with HPLC (Waters, Milford, MA) using a C 18 Luna column (4.6×150 mm; particle size 5 µm; Phenomenex, Torrance, CA). Samples (20 µl) were injected into the column, and gradient elution was used for fractional separation with two solvents at a flow of 1 mL/minute, Solvent A consisted of 10% Methanol in H_2_O adjusted pH 3.5 with formic acid0. Solvent B consisted of 20% water (pH 3.5), 20% methanol and 60% acetronitrile. The gradient consisted of 0 minute at 100% A, followed by 5 minutes at 85% A, 10 minutes at 80% A, 20 minutes at 75% A, 25 minutes at 73% A27 minutes at 60% A, 30 minutes at 60% A, 35 minutes at 50% A, 40 minutes at 10% A, 45 minutes at 0% A, and 50 minutes at 100% A and holding at 100% A for a final 55 minutes. Five minutes of equilibration at 100% A was performed before and after each injection.

### *In vitro* hydrogen peroxide production

Fungal cultures were grown on PDA medium and incubated in two different temperatures (25°C and 30°C) and light conditions (dark and light). Accumulation of hydrogen peroxide was evaluated after 5 days of growth following method described by Munkres (1990) [43]. In brief, hydrogen peroxide was indicated by the accumulation of a red precipitate after being flooded with 5 ml solution of 100 mM potassium phosphate buffer, pH 6.9, 2.5 mM diaminobenzidine tetrachloride and 5 purpurogallin units/ml of horseradish peroxidase (Type VI, Sigma Chemical Company, St. Louis, MO). Plates were incubated at 30°C in dark for one hour before the solution was discarded. Afterward, plates were inverted and incubated at 30°C in the dark for 24 h and then photographed.
